# Full Anatomical Recovery of the Dopaminergic System after a Complete Spinal Cord Injury in Lampreys

**DOI:** 10.1155/2015/350750

**Published:** 2015-03-24

**Authors:** Blanca Fernández-López, Daniel Romaus-Sanjurjo, María Eugenia Cornide-Petronio, Sonia Gómez-Fernández, Antón Barreiro-Iglesias, María Celina Rodicio

**Affiliations:** Department of Cell Biology and Ecology, CIBUS, Faculty of Biology, University of Santiago de Compostela, 15782 Santiago de Compostela, Spain

## Abstract

Following a spinal injury, lampreys at first are paralyzed below the level of transection. However, they recover locomotion after several weeks, and this is accompanied by the regeneration of descending axons from the brain and the production of new neurons in the spinal cord. Here, we aimed to analyse the changes in the dopaminergic system of the sea lamprey after a complete spinal transection by studying the changes in dopaminergic cell numbers and dopaminergic innervation in the spinal cord. Changes in the expression of the D2 receptor were also studied. We report the full anatomical regeneration of the dopaminergic system after an initial decrease in the number of dopaminergic cells and fibres. Numbers of dopaminergic cells were recovered rostrally and caudally to the site of injury. Quantification of dopaminergic profiles revealed the full recovery of the dopaminergic innervation of the spinal cord rostral and caudal to the site of injury. Interestingly, no changes in the expression of the D2 receptor were observed at time points in which a reduced dopaminergic innervation of the spinal cord was observed. Our observations reveal that in lampreys a spinal cord injury is followed by the full anatomical recovery of the dopaminergic system.

## 1. Introduction

In humans, complete spinal cord injuries cause an irreversible loss of function below the site of lesion and lead to permanent disability. This is mainly due to the absence of regeneration of descending axons and the failure of replacement of damaged spinal neurons. In contrast, lampreys spontaneously recover locomotion after a complete spinal cord injury [1]. During the recovery process lampreys are able to regenerate axotomized descending axons [2–4] and produce new spinal neurons [5]. Recent studies have also shown that different neurotransmitters systems adapt and show plastic changes after a spinal cord injury in lampreys, which could also contribute to the recovery of function (serotonergic system: [6, 7]; aminoacidergic systems: [8–10]). So, both regeneration and plasticity events appear to contribute to the spontaneous recovery of function in lampreys. It is of great biological interest to study the amazing response of lampreys to a spinal cord injury and specifically how the different neurotransmitter systems react to the injury.

The spinal cord receives inputs from different neurotransmitter systems located in the brain. Among them, the monoaminergic systems (i.e., dopaminergic, serotonergic, and noradrenergic systems) play an important role in the modulation of spinal locomotor circuits. Historically, more efforts have been put on the study of the serotonergic spinal system than on the dopaminergic one. However, across different species dopamine has profound and diverse effects on rhythmically active motor networks (for a recent review see [11]). In lampreys, dopamine elicits a complex modulatory effect on swimming behavior. Low dopamine concentrations (0.1–10 *μ*M) result in an increase in locomotor frequency, while higher concentrations (10–100 *μ*M) slow the rhythm, and concentrations of 100 *μ*M–1 mM can suppress the rhythm [12–14]. The increase in locomotor frequency can be reproduced in freely swimming animals [15] and appears to be mediated by D2 receptors [12]. Concentration dependent dopamine effects are also observed in tadpoles, where dopamine reduces locomotor activity via D2-like mechanisms at low concentrations (2 *μ*M) and promotes locomotor activity at higher concentrations (50 *μ*M) via D1-like mechanisms [16]. In mammals, dopamine is able to promote locomotion when introduced to the intact animal [17].* In vitro* preparations of fictive locomotion of the neonatal rodent spinal cord have shown that dopamine can activate fictive locomotor activity in rats [18, 19], but not in mice, although D1 agonist alone can be sufficient for locomotion in mice [20]. In mammals, dopamine may be promoting ongoing locomotor activity through the activation of D1-like receptors, while the slowing effect of dopamine on fictive locomotor frequency is through D2-like receptor mechanisms (for a review see [11]). Previous research shows that dopamine plays an important role in the modulation and control of locomotion in vertebrates, from lampreys to mammals.

Interestingly, recent reports have also shown the importance of dopamine signalling not only during normal locomotion, but also in the recovery of locomotion following spinal cord injury in mammals [21–23] or of dopamine as a signal controlling spinal cord development and regeneration in zebrafish [24, 25]. This reveals that dopamine could also be a key player for regeneration and the recovery of function after spinal cord injury in vertebrates.

Here, we report a study of the anatomical changes that occur in the dopaminergic system of lampreys after a complete spinal cord injury and during the recovery period by studying: (1) the changes in the dopaminergic innervation of the spinal cord, (2) the changes in dopaminergic cell numbers, and (3) the changes in the expression of the dopamine D2 receptor in the spinal cord. We aimed to investigate if lampreys recover or adapt the dopaminergic system during the process of functional recovery after a complete spinal cord injury and compare this process to other regenerating and nonregenerating vertebrates. The source of spinal dopamine in mammals is the descending hypothalamic projection [26], which is also present in lampreys [27] and all the other vertebrates [26]. This high degree of conservation facilitates a comparison between lampreys and other vertebrates in terms of recovery of the dopaminergic innervation of the spinal cord after an injury. However, it is also of interest that lampreys, as opposed to other vertebrates, have intrinsic dopaminergic cells in the spinal cord. So, our study also offers a new model to study the spontaneous recovery of dopaminergic cells after a traumatic injury in vertebrates.

## 2. Material and Methods

### 2.1. Animals

Mature and developmentally stable larval sea lampreys (*Petromyzon marinus* L., >100 mm body length, 4 to 7 years of age) were used for the present investigation. Larval sea lampreys were collected from the river Ulla (Galicia, Spain) with permission from the Xunta de Galicia and maintained in aerated fresh water aquaria at 16°C with a bed of river sediment until their use for experimental procedures. All animal experimental procedures were approved by the Bioethics Committee at the University of Santiago de Compostela and complied with Spanish and European guidelines for the care and use of animals in research.

### 2.2. Complete Spinal Cord Transection

Before surgery, animals were deeply anaesthetized by immersion in 0.1% tricaine methanesulfonate (MS-222; Sigma, St. Louis, MO). A complete transection of the spinal cord was performed with a scalpel at the level of the 5th gill as previously described [4, 7]. Animals (*n* = 33) were allowed to recover for 2 (*n* = 13), 4 (*n* = 10), 10 (*n* = 5), or 24 (*n* = 5) weeks after lesion (wpl). Visual observations indicated that normal appearing swimming behaviour was present in the 5 animals processed at 24 wpl. Control unlesioned animals (*n* = 12) were always processed in parallel with lesioned animals.

### 2.3. Dopamine Immunohistochemistry

Dopamine immunofluorescence experiments were performed in 14 *μ*m transverse cryostat spinal cord sections of control and lesioned animals using a rabbit anti-dopamine antibody (1 : 750; Dr. Steinbusch, Maastricht University) and as previously described [28]. Ten spinal cord hemisections (one spinal cord side) were photographed 450 *μ*m both rostrally and caudally to the site of injury with the confocal spectral microscopes TCS-SP2 and SP5 (Leica, Wetzlar, Germany). In control animals, 20 sections at the level of the 5th gill were photographed. One out of three consecutive sections was photographed in unlesioned and lesioned (rostral and caudal) animals. The semiautomatic quantification of the number of dopamine immunofluorescent profiles per hemisection (the hemisection was randomly chosen for each section) was done using ImageJ and as previously described for the quantification of monoaminergic or aminoacidergic positive profiles in spinal cord transverse sections of zebrafish and lampreys [9, 29, 30].

### 2.4. Cell Counts

Two types of dopaminergic spinal cells were quantified: ventromedial (VM) and cerebrospinal fluid-contacting (CSFc) cells. The number of cells in the rostral and caudal spinal cord was obtained from stereological counts of stacks of confocal images from the 10 spinal cord sections rostral and 10 caudal to the lesion site (1 out of 3 consecutive sections) as previously described [30]. Stereological counting was performed discarding the cells located in the first optical section of the confocal stack of each spinal cord section. The number of cells in 450 *μ*m was estimated from the number of cells counted in each spinal section and then the mean number of cells in 450 *μ*m was calculated for each animal from the results of the 10 sections separately counted for the rostral and caudal spinal cord.

### 2.5. *In Situ* Hybridization

*In situ* hybridization for the detection of dopamine D2 receptor transcripts was performed in cryostat transverse spinal cord sections of control unlesioned and spinal transected animals.* In situ* hybridization experiments were performed as previously described for riboprobes against the serotonin 1a receptor (5-ht1a) [31]. The riboprobe against the sea lamprey D2 receptor transcript was generated by using the following primers 5′-GTGCCCTCTTCTCTTTGGCT-3′ (forward) and 5′-TAAGCTTTAATACGACTCACTATAGGGAGAAAGAAGGGCATCCAGCAGAC-3′ (reverse). These primers were generated based on the river lamprey (*Lampetra fluviatilis*) D2 receptor mRNA sequence deposited in the NCBI GenBank (GenBank accession number: HQ331119 [31]). The reverse primer includes the sequence of the T7 promoter for riboprobe generation. Digoxigenin- (DIG-) labeled riboprobes were synthesized using the amplified fragments as templates and following standard protocols using T7 polymerase (Roche Diagnostics, Germany).

As previously shown by others, D2* in situ* signal appears as a dotted labelling in sections of the lamprey central nervous system [32, 33]. The semiautomatic quantification of the number of these positive D2 profiles per spinal cord hemisection was done as previously described for the quantification of 5-ht1a positive profiles, which also produced a dotted* in situ* labelling [7].

### 2.6. Statistical Analyses

For statistical analysis, the program Prism (GraphPad Software, La Jolla, CA) was used. Variability of values is always given as SEM. Normality was analysed with the Kolmogorov-Smirnov normality test. Data sets that passed the normality test were analysed for statistical significance with a One-Way ANOVA. Data sets that did not pass the normality test were analysed for statistical significance with the Kruskal-Wallis One-Way ANOVA for nonnormally distributed data. Bonferroni's or Dunn's multiple comparison tests were used to compare pairs of data.

## 3. Results

### 3.1. Dopamine Immunoreactivity in Unlesioned Control Animals

**Figure 1:**
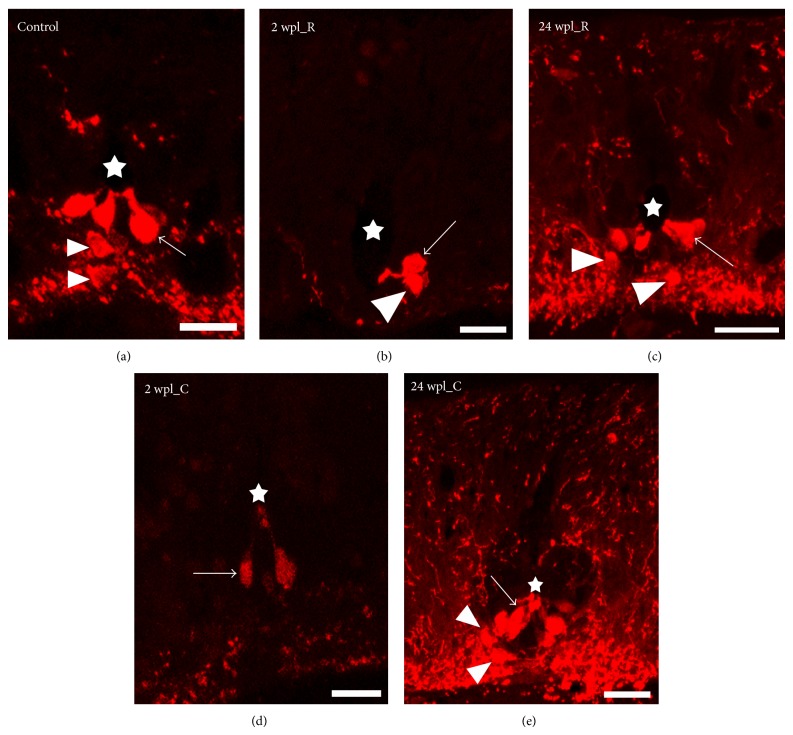
Confocal photomicrographs of transverse sections of the spinal cord showing details of the dopaminergic cells in control and lesioned animals. (a) Dopaminergic cells in a control unlesioned larva. ((b), (d)) Dopaminergic cells in a 2 wpl larva, rostral (b) and caudal (d) to the site of injury. ((c), (e)) Dopaminergic cells in a 24 wpl larva, rostral (c) and caudal (e) to the site of injury. CSFc cells (arrows), VM cells (arrowheads), star points to the central canal. In all photomicrographs dorsal is at the top. Scale bars = 25 *μ*m.

At the level of the 5th gill the spinal cord of mature larval sea lamprey shows the presence of two main types of dopamine immunoreactive (-ir) cells: CSFc cells that are located in the ventral portion of the central canal and non-CSFc cells that are located in the ventromedial region of the spinal cord (VM cells) (Figure 1(a)). In addition, very scarce dopamine-ir cells were occasionally observed in the lateral or dorsal grey (not shown). Here, we analysed the changes in the number of cells for the two main dopamine-ir populations, CSFc and VM cells, during spinal cord regeneration after a complete spinal cord transection.

**Figure 2:**
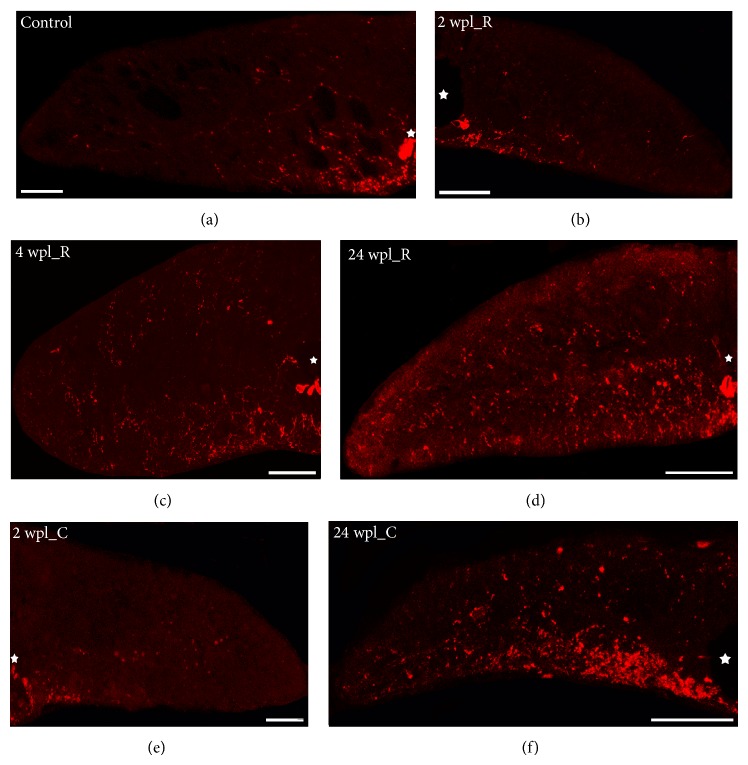
Confocal photomicrographs of transverse sections of the spinal cord showing details of the dopaminergic innervation of the spinal cord in control and lesioned animals. (a) Dopamine-ir fibers in a control unlesioned larva. (b) Dopamine-ir fibers in the rostral stump of a 2 wpl larva. (c) Dopamine-ir fibers in the rostral stump of a 4 wpl larva. (d) Dopamine-ir fibers in the rostral stump of a 24 wpl larva. (e) Dopamine-ir fibers in the caudal stump of a 2 wpl larva. (f) Dopamine-ir fibers in the caudal stump of a 24 wpl larva. Star indicates the central canal. In all microphotographs dorsal is at the top. Scale bars = 50 *μ*m.

At this spinal cord level, dopamine-ir fibres are observed mainly in the ventromedial region of the white matter, below the giant reticulospinal axons, forming a dense plexus of varicose fibres (Figures 1(a) and 2(a)). This plexus appears to be constituted primarily by processes of the VM dopamine-ir cells [13]. Dopamine-ir fibres are also observed in the lateral column and in the dorsal region of the white matter (Figure 2(a)). The distal processes of the dopamine-ir CSFc cells can be traced to the lateral cell column, the ventral area of the dorsal column, and to the ventromedial region of the cord [34]. Dopaminergic spinal-projecting neurons have been also reported in the brain of the sea lamprey [27]; therefore, some of the dopamine-ir fibres at this spinal cord level probably belong to the descending dopaminergic projections.

### 3.2. D2 Receptor Expression in Unlesioned Animals

**Figure 3:**
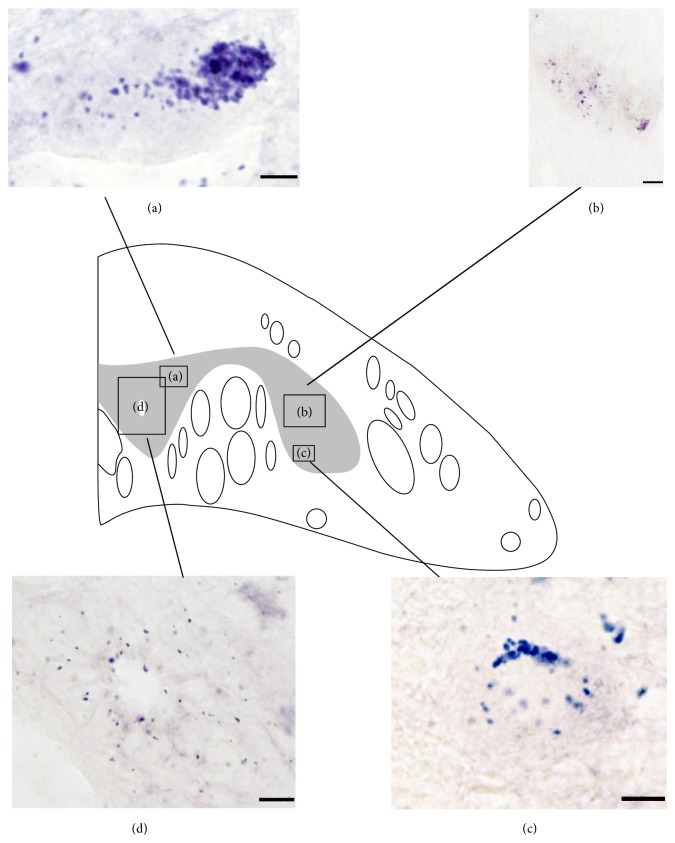
Expression of the D2 receptor in control animals. (a) Dorsal cell showing expression of the D2 transcript in its soma. (b) Interneurons showing expression of D2 transcript. (c) Expression of D2 transcripts in CSFc and ependymal glial cells around the central canal. (d) Expression D2 transcripts in spinal motor neurons. In all microphotographs dorsal is at the top. Scale bars = 50 *μ*m.

At the level of the 5th gill, D2 transcript expression was observed in most of the spinal cord neurons: dorsal cells, motor neurons, and interneurons (Figure 3). No expression of the D2 transcript was observed in edge cells. The dorsal cells showed a group of positive dots in their soma (Figure 3(a)). D2 transcript expression was observed in interneurons of different sizes and morphologies located throughout the dorsal and lateral grey matter (Figure 3(b)). Expression of the D2 transcript was also observed in cells that were identified as spinal motor neurons based on their morphology and location in the spinal motor column (Figure 3(c)). Finally, expression of D2 transcripts was also observed in cells surrounding the central canal (CSFc and ependymal glial cells) with granules of positive expression in their somas and in the dendrites of CSFc cells (Figure 3(d)). The expression of the D2 transcript has also been recently described in the rostral spinal cord of the adult river lamprey [33], and the pattern of expression appears to be very similar between the larval sea lamprey (present results) and the adult river lamprey [33].

### 3.3. Dopaminergic Cell Numbers Are Recovered after a Complete Spinal Cord Transection

**Figure 4:**
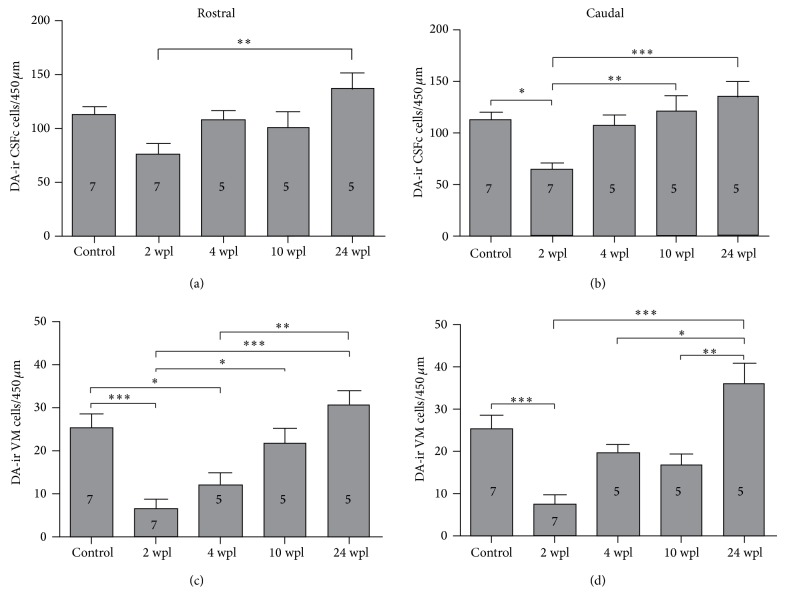
Dopaminergic cell numbers are recovered after a complete spinal cord injury. (a) Quantification of CSFc dopamine-ir cells in the rostral spinal cord (ANOVA, *P* = 0.0049 with Bonferroni's posttest, ^***^ *P* < 0.0001). (b) Quantification of CSFc dopamine-ir cells in the caudal spinal cord (ANOVA, *P* = 0.0006 with Bonferroni's posttest, ^*^ *P* < 0.05, ^**^ *P* < 0.01, and ^***^ *P* < 0.0001). (c) Quantification of VM dopamine-ir cells in the rostral spinal cord (ANOVA, *P* < 0.0001 with Bonferroni's posttest, ^*^ *P* < 0.05, ^**^ *P* < 0.01, and ^***^ *P* < 0.0001). (d) Quantification of VM dopamine-ir cells in the caudal spinal cord (ANOVA, *P* < 0.0001 with Bonferroni's posttest, ^*^ *P* < 0.05, ^**^ *P* < 0.01, and ^***^ *P* < 0.0001).

The changes in dopamine-ir cell numbers are shown in Figures 1 and 4. Rostral to the site of injury, at 2 wpl, we found a 31.7% (nonsignificant) lower number of CSFc dopamine-ir cells as compared to unlesioned animals. At 24 wpl the number of CSFc dopamine-ir cells rostral to the site of injury was significantly increased to 121.6% as compared to 2 wpl and was not significantly different to unlesioned animals (Figures 1 and 4(a)).

Caudal to the site of injury, at 2 wpl, we found a significant 42.5% lower number of CSFc dopamine-ir cells as compared to unlesioned animals. At 24 wpl the number of CSFc dopamine-ir cells caudal to the site of injury was significantly increased to 120.9% as compared to 2 wpl and was not significantly different to unlesioned animals. The number of CSFc dopamine-ir cells caudal to the site of injury was already significantly increased to 106.9% at 10 wpl as compared to 2 wpl (Figures 1 and 4(b)).

Rostral to the site of injury, at 2 and 4 wpl, we found a significant 73.5% and 51.9% lower number of VM dopamine-ir cells, respectively, as compared to unlesioned animals. At 24 wpl the number of VM dopamine-ir cells rostral to the site of injury was significantly increased to 119.4% as compared to 2 and 4 wpl and was not significantly different to unlesioned animals. The number of VM dopamine-ir cells rostral to the site of injury was already significantly increased to 85.1% at 10 wpl as compared to 2 wpl (Figures 1 and 4(c)).

Caudal to the site of injury, at 2 wpl, we found a significant 70.8% lower number of VM dopamine-ir cells as compared to unlesioned animals. At 24 wpl the number of VM dopamine-ir cells caudal to the site of injury was significantly increased to 141.2% as compared to 2 wpl and was not significantly different to unlesioned animals (Figures 1 and 4(d)).

### 3.4. The Dopaminergic Innervation of the Spinal Cord Is Recovered after a Complete Spinal Cord Transection

**Figure 5:**
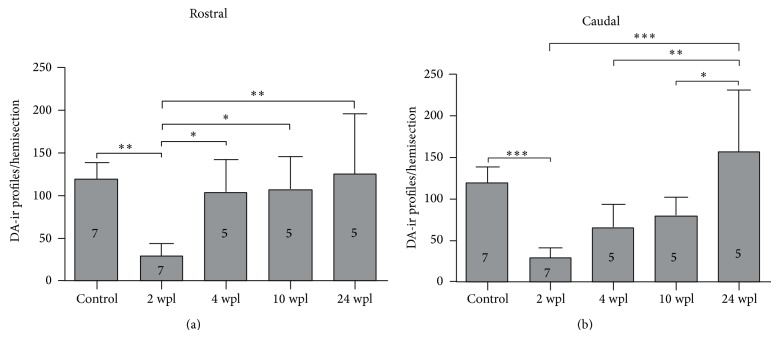
Dopaminergic profile numbers are recovered after a complete spinal cord injury. (a) Quantification of dopaminergic profiles in the rostral spinal cord (ANOVA, *P* = 0.0009 with Bonferroni's posttest, ^*^ *P* < 0.05, ^**^ *P* < 0.01, and ^***^ *P* < 0.0001). (b) Quantification of dopaminergic profiles in the caudal spinal cord (ANOVA, *P* < 0.0001 with Bonferroni's posttest, ^*^ *P* < 0.05, ^**^ *P* < 0.01, and ^***^ *P* < 0.0001).

The changes in the dopamine-ir innervation of the spinal cord are shown in Figures 2 and 5. Rostral to the site of injury, at 2 wpl, we found a significant 74.8% lower number of dopaminergic profiles per spinal cord hemisection as compared to unlesioned animals. At 24 wpl the number of dopaminergic profiles per spinal cord hemisection rostral to the site of injury was significantly increased to 105.3% as compared to 2 wpl and was not significantly different to unlesioned animals. The number of dopaminergic profiles per spinal cord hemisection rostral to the site of injury was already significantly increased to 86.8% and 89.8% at 4 wpl and 10 wpl, respectively, as compared to 2 wpl (Figures 2 and 5(a)).

Caudal to the site of injury, at 2 wpl, we found a significant 75.4% lower number of dopaminergic profiles per spinal cord hemisection as compared to unlesioned animals. At 24 wpl the number of dopaminergic profiles per spinal cord hemisection rostral to the site of injury was significantly increased to 131.6% as compared to 2 wpl and was not significantly different to unlesioned animals (Figures 2 and 5(b)).

### 3.5. The Spinal Cord Injury and a Reduced Dopaminergic Innervation Do Not Affect the Expression of the D2 Receptor Transcript in the Sea Lamprey Spinal Cord

**Figure 6:**
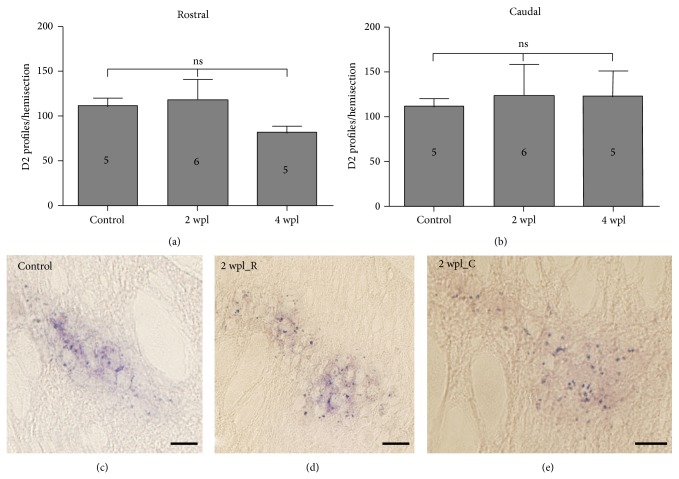
D2* in situ* profile numbers do not change after a complete spinal cord injury. (a) Quantification of D2 profiles in the rostral spinal cord. (b) Quantification of D2 profiles in the caudal spinal cord. (c) Photomicrograph showing the D2 receptor expression in the spinal cord of a control larva. (d) Photomicrograph showing the D2 receptor expression in the rostral spinal cord of a 2 wpl larva. (e) Photomicrograph of the D2 receptor expression in the caudal spinal cord of a 2 wpl larva. In all microphotographs dorsal is at the top. Scale bars = 50 *μ*m.

In a previous study we identified acute changes in the expression of 5-ht1a transcripts after a complete spinal cord transection in the larval sea lamprey [7]. Here, we quantified the number of D2 positive* in situ* profiles in hemisections of the spinal cord, both rostral and caudal to the lesion site at 2 and 4 wpl, and in unlesioned larvae (Figure 6). Compared to control unlesioned values, no significant differences were observed in the number of D2 profiles per hemisection in the rostral (One-Way ANOVA, *P* = 0.2490) or caudal (Kruskal-Wallis ANOVA, *P* = 0.6210) stumps of the spinal cord at 2 or 4 wpl (Figure 6). This indicated that the complete spinal cord injury and/or the initial decrease (at 2 to 4 wpl) in dopaminergic fibres and cells do not induce changes in the expression of D2 receptor transcripts in the sea lamprey.

## 4. Discussion

Our study shows that a high and complete spinal cord injury in lampreys causes initial decrease in the number of dopaminergic cells and fibres in the spinal cord, which is followed by the full anatomical recovery of the dopaminergic spinal system. This is in contrast to the glutamatergic system, which is not fully recovered 6 months after a complete spinal cord injury in lampreys, even in the presence of functional recovery [10]. Both the dopaminergic innervation of the spinal cord (rostral and caudal to the site of injury) and the number of dopaminergic intrinsic spinal cells are fully recovered 6 months after the complete spinal cord injury. These results indicate that, in lampreys, each neurotransmitter system responds differently to the spinal cord injury to achieve functional recovery. The importance of each of these changes and the interaction between the different neurotransmitter spinal systems (i.e., metaplasticity) for the recovery of function should be analysed in future studies. The analysis of our present observations points to several interesting facts that we discuss here.

When looking at changes in the number of cells, we observed a faster recovery of the number of CSFc dopamine-ir cells than that of VM dopamine-ir cells after the complete spinal cord injury. Although we cannot assure that these are newly generated cells, the fact that the number of VM cells is recovered later could reflect the time needed for the differentiation and migration of these cells after regeneration from ependymal progenitor cells located around the central canal. Several types of interneurons as well as motor neurons of adult zebrafish, another regenerating vertebrate, are regenerated after a complete spinal cord injury from ependymoradial glial cells located around the central canal [25, 30, 35–37]. A recent study has also reported the production of new neurons after a complete spinal cord injury in lampreys based on the colocalization of BrdU and the neuronal marker Hu [5]. Present and past results suggest that dopaminergic cells could be newly generated after the injury in lampreys. Interestingly, Zhang and coworkers [5] reported that in lampreys the newly generated neurons where observed only close to the central canal with no newly generated neurons migrating away from it up to 5 wpl, which would explain the extra time needed by VM cells to recover. Further studies should attempt to demonstrate if the recovery of dopaminergic cell numbers is due to the production of new cells and whether these have their origin in ependymoradial glial progenitors. Our study provides a new and interesting model to investigate the spontaneous regeneration of dopaminergic cells after a traumatic injury in vertebrates.

Following the complete spinal cord injury, we observed an initial loss of rostral innervation, not just caudally as one could expect. Obviously, the loss of rostral intrinsic interneurons and their corresponding fibres contributes to this process. However, this might be also related to the dieback of damaged dopaminergic fibres, a process known to occur in axotomized descending axons of lampreys [38]. The initial dieback of rostral dopaminergic fibres has been also reported in adult zebrafish after a complete spinal cord injury [36]. Interestingly, adult zebrafish have no intrinsic dopaminergic cells in the spinal cord [36], which suggests that the reduced rostral innervation after the injury in lampreys could also be explained, at least partially, by a dieback process.

An important difference between the observations in lampreys and zebrafish is that we did not detect a significant increase in the innervation of the rostral spinal cord up to 24 wpl compared to the control situation, instead the number of profiles reached levels similar to control values. In adult zebrafish, dopaminergic fibres sprout after the initial dieback and a significant increased innervation of the rostral spinal cord was observed 13 weeks after the injury [36]. This rostral sprouting leading to increased innervation has been also reported for serotonergic fibres in lampreys [6] and zebrafish [36, 39]. So, our study reveals interesting and differential feature of the behaviour of dopaminergic fibres after a complete spinal cord injury in lampreys, again stressing the importance of studying this phenomenon in different vertebrate models of spinal cord injury.

Here, we also report for the first time the spontaneous and complete recovery of the dopaminergic innervation of the spinal cord distal to the site of injury in any vertebrate species. The fact that the recovery of the caudal innervation occurred later than that of the rostral spinal cord indicates that part of the distal reinnervation could be due to the regrowth of descending fibres through the site of injury. The full reinnervation of the caudal spinal cord was not observed in another regenerating vertebrate like adult zebrafish [36], even in the presence of behavioural recovery. Our results show that lampreys are more efficient than jawed vertebrates in the recovery of the dopaminergic innervation distal to the site of injury. Future studies should confirm if the caudal recovery is due to the regrowth of descending fibres or due to compensation by increased innervation from intrinsic neurons.

The absence of changes in the expression of the D2 receptor after the complete spinal cord injury is also of interest. We have previously reported an acute increase in the expression of the 5-ht1a after a complete spinal cord injury in lampreys [7]. An increase in the expression of the D4 receptor has been observed following a complete spinal cord injury in adult zebrafish [25]. Our study indicates that different monoaminergic receptors respond in different ways to achieve recovery in regenerating vertebrates and that there is not just a general acute increase in the expression of these receptors due to the initial decrease in monoaminergic innervation. In zebrafish, the D4 receptor is known to mediate the promoting effect of dopamine on motor neuron regeneration [25]. Interestingly, a recent study using a toxin ablation paradigm has shown that dopamine inhibits the production of new dopaminergic cells in the midbrain of salamanders and that this effect could be mediated by D2 receptors [40]. The lack of changes in the expression of the D2 receptor suggests that it might not play a crucial role during spinal cord regeneration. In any case, our study offers the opportunity to investigate the role of the D2 receptor during the regeneration of dopaminergic cells after a traumatic injury in lampreys.

## 5. Conclusions

Dopaminergic drugs have been proven effective to promote locomotion in mammalian models of spinal cord injury [21–23, 41]. Here, we have shown that larval sea lampreys spontaneously recover their spinal dopaminergic system after a complete spinal cord injury. This model will set the basis for finding molecules implicated in the intrinsic and spontaneous regeneration of dopaminergic cells and fibres in vertebrates. In addition, our anatomical study will help future functional studies on the role of dopamine in the control of locomotion after recovery from a spinal cord injury.
